# Smoking cessation prior to elective plastic surgery: why, when and how?

**DOI:** 10.1186/1617-9625-12-S1-A18

**Published:** 2014-06-06

**Authors:** Vasileios Theocharidis, Konstantinos P Economopoulos

**Affiliations:** 1 Society of Junior Doctors, Athens, 15123, Greece; 2 Medical school, National and Kapodistrian University of Athens, Athens, 115 27, Greece; 3 Department of Surgery, Massachusetts General Hospital, Harvard Medical School, Boston, Massachusetts, 02114, USA

## Background

Tobacco is a serious nuisance in plastic surgery, mainly through the effects of its inhaled constituents on wound healing physiology. While there are thousands of chemical compounds in the inhaled smoke, the most prominent ones are nicotine, carbon monoxide and hydrogen cyanide, which are partially responsible for impaired wound healing through decreased oxygen delivery and utilization, and deranged collagen deposition [1]. Our aim is to summarize the existing literature on the effects of smoking in various plastic surgical procedures, and on the correct timing and available methods of preoperative smoking cessation.

## Materials and methods

A literature review of PubMed was performed. The inclusion criteria were: articles on the effects of smoking on wound healing, plastic surgery and hand surgery, on the timing of preoperative smoking cessation and on methods of smoking cessation. The search terms used in combinations were “smoking”, “plastic surgery”, “wound healing”, “hand surgery”, “smoking cessation”.

## Results

In facelift procedures, skin slough is an important complication and there is a strong association between smoking and higher skin slough rates [2]. A similar relationship can be identified in abdominoplasty, as smokers suffer from a higher percentage of wound healing complications [3]. Complications like T-junction necrosis and infections were also higher in smokers who underwent breast reduction [4]. Regarding breast reconstruction, two studies showed higher rates of reconstructive failure and overall complications in smokers [5]. However, no significant difference was found regarding flap loss and vascular thrombosis between smokers and non-smokers [6]. In microsurgical free flap transfer, the relationship visited above is prevalent, with higher wound healing-related complication rates for smokers. [7]. In hand surgery, it has been shown that smoking post-replantation decreases digital blood flow significantly [8]. A meta-analysis showed the detrimental effect of smoking on the success rate of digit replantation (61.1% in smokers vs. 96.7% in non-smokers) [9]. While there is no consensus regarding the optimal smoke-free period preoperatively, many authors agree that 4 weeks are capable of reducing the rates of smoking-related complications satisfactorily. Some authors support the use of cotinine measurement, as patient history can prove notoriously inaccurate [10]. In order to improve the low cessation rate achieved by no intervention (about 1 in 8) [11], nicotine replacement therapy and medication can be used effectively [12].

## Conclusions

Smoking is an independent risk factor for wound healing complications, but not for free flap loss. Smokers who are candidates for plastic surgery should cease smoking for an adequate amount of time preoperatively. Nicotine replacement therapy and medication are effective in assisting their smoking cessation efforts.
